# Supplementary material for: Functional and ecomorphological evolution of orbit shape in mesozoic archosaurs is driven by body size and diet
Source: Commun Biol. 2022 Aug 11;5:754. doi: 10.1038/s42003-022-03706-0 (PMC9372157; doi:10.1038/s42003-022-03706-0)
Supplement: Supplementary file 2 — Supplementary Information [file 42003_2022_3706_MOESM2_ESM.pdf]

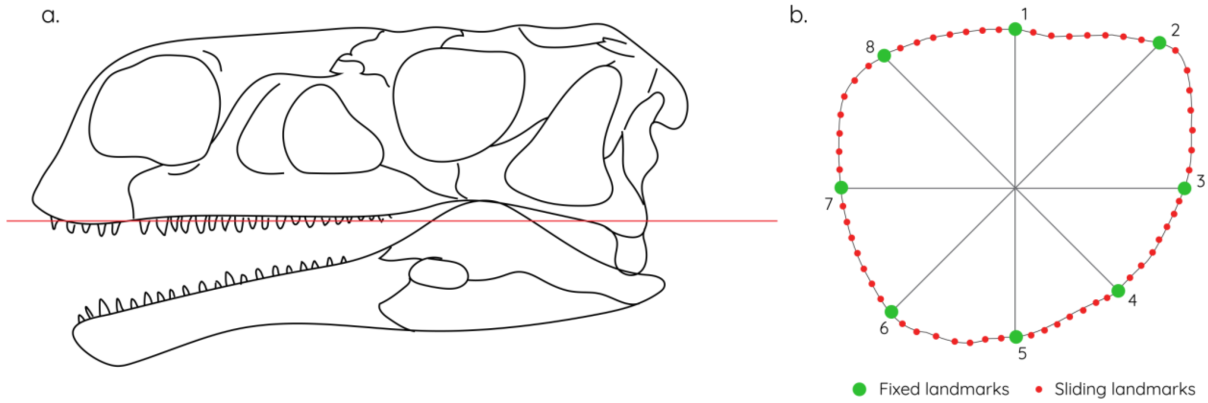

Supplementary figure. 1 | Digitisation of orbit morphology. (**a**) Skull aligned with maxillary tooth row parallel to horizontal plane. (**b**) Selection of eight fixed and 56 semi-landmarks.

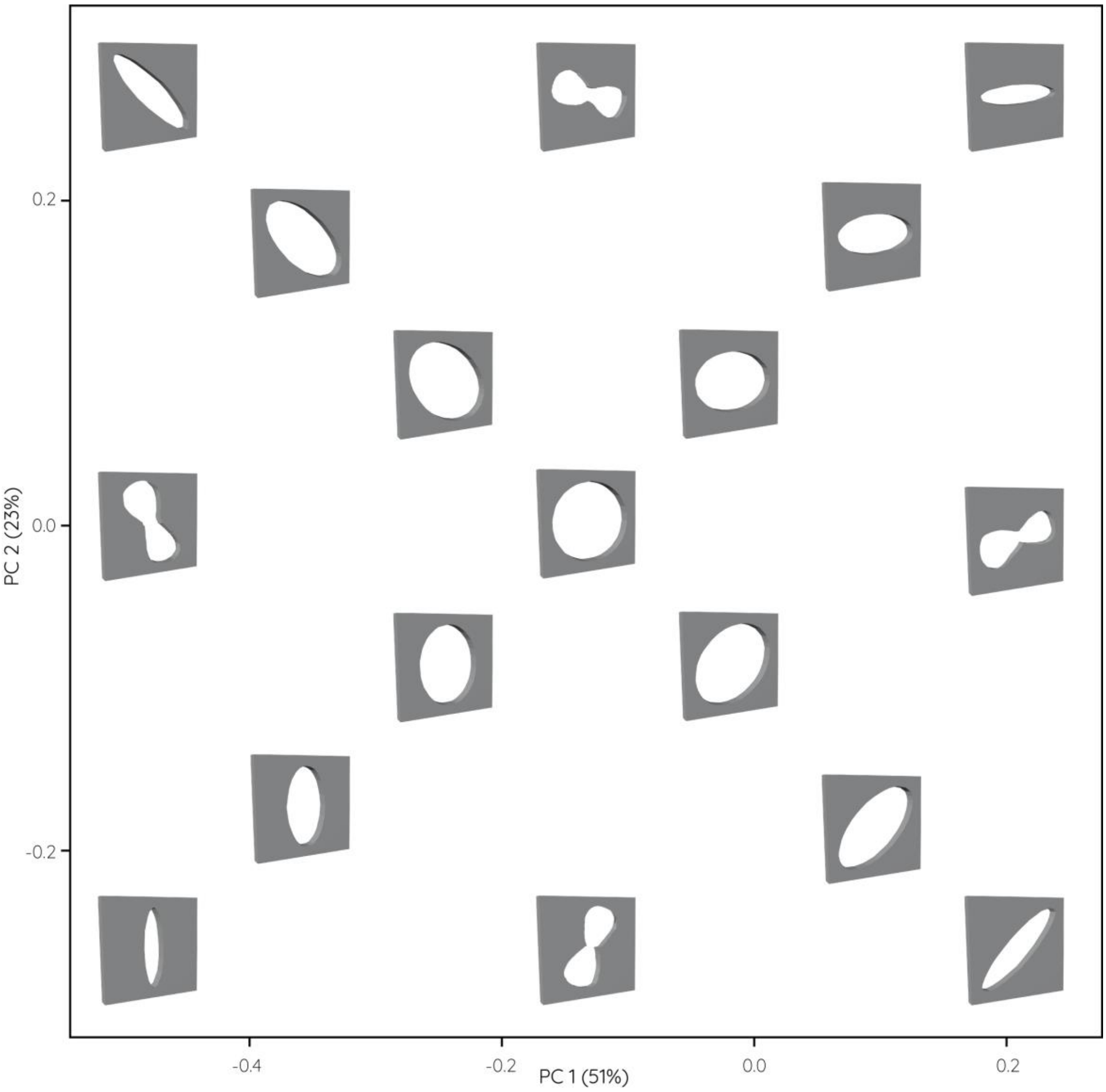

Supplementary figure. 2 | Theoretical flat-plate models. Position of models based on landmark-based shape quantification.

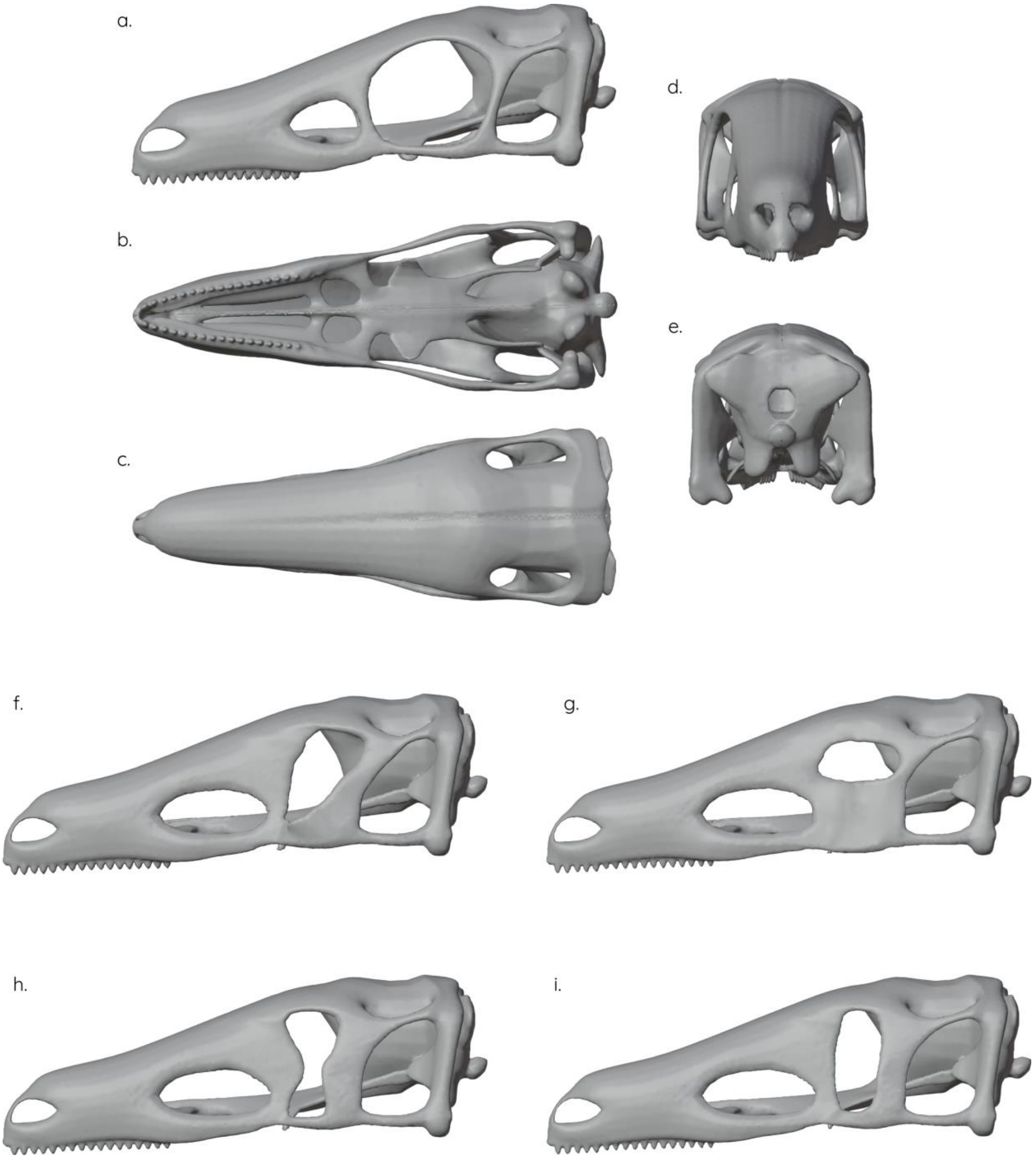

Supplementary figure. 3 | Hypothetical skull models with different orbit shapes. Base skull model in (**a**) lateral, (**b**) ventral, (**c**) dorsal, (**d**) anterior, and (**e**) posterior view. Models with (**f**) wedge-shaped, (**g**) dorsoventrally compressed, (**h**) keyhole-sahped, and (**i**) elliptical orbit shape.

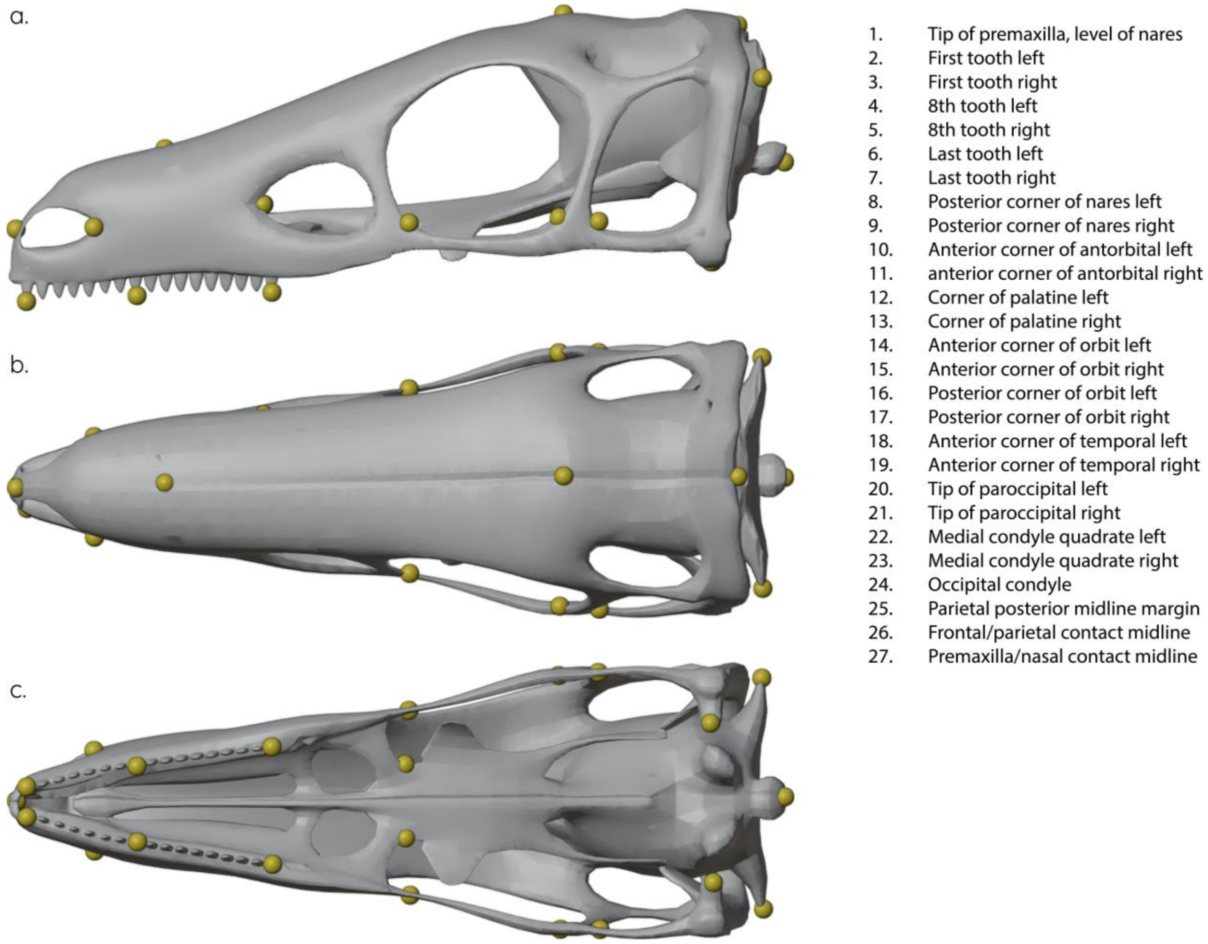

Supplementary figure. 4 | Placement of landmarks for deformation models. Skull model in (**a**) lateral, (**b**) dorsal, and (**c**) ventral view.

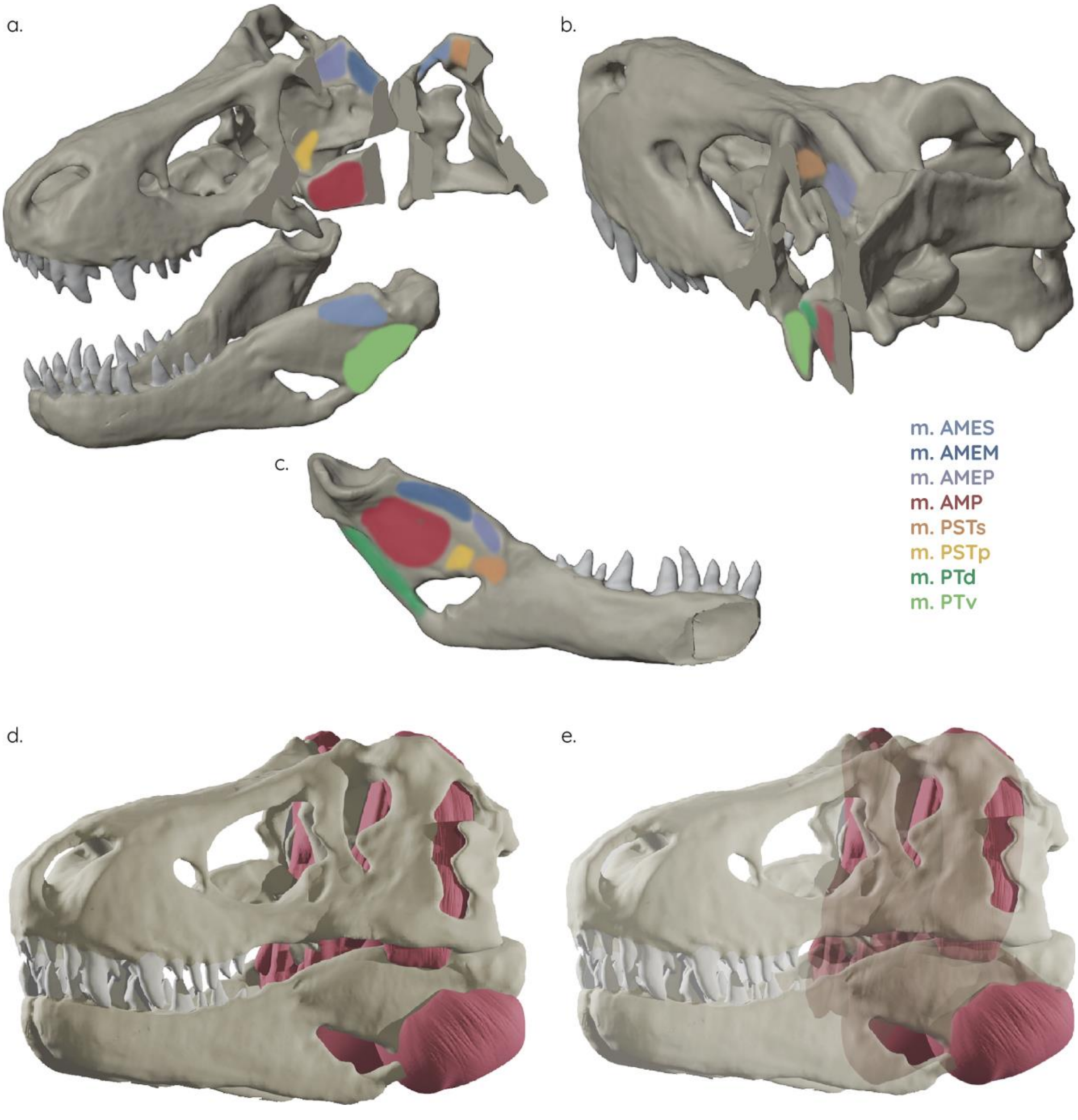

Supplementary figure. 5 | Reconstructed jaw adductor musculature of *Tyrannosaurus rex*. Muscle attachment sites mapped onto the skull in (**a**) oblique lateral, and (**b**) oblique dorsal view, and onto the (**c**) mandible in medial view. Three-dimensional reconstructions with the skull rendered (**d**) solid and (**e**) transparent. Abbreviations: m. AMEM, m. adductor mandibulae externus medialis; m. AMEP, m. adductor mandibulae externus profundus; m. AMES, m. adductor mandibulae externus superficialis; m. AMP, m. adductor mandibulae posterior; m. PSTp, m. pseudotemporalis profundus; m. PSTs, m. pseudotemporalis superficialis; m. PTd, m. pterygoideus dorsalis; m. PTv, m. pterygoideus ventralis.

**Muscle**	**m. AMES**	**m. AMEM**	**m. AMEP**	**m. AMP**	**m. PSTs**	**m. PSTp**	**m. PTd**	**m. PTv**
**Force [N]**	**2809**	**2262**	**1620**	**2365**	**475**	**469**	**2018**	**5835**
**Total [N]**	**17853**							

Supplementary table. 1 | Jaw adductor muscle forces for *Tyrannosaurus rex* as used in the biomechanical analyses. Muscle force values for single side only.
