# Supplementary material for: Functional and ecomorphological evolution of orbit shape in mesozoic archosaurs is driven by body size and diet
Source: Commun Biol. 2022 Aug 11;5:754. doi: 10.1038/s42003-022-03706-0 (PMC9372157; doi:10.1038/s42003-022-03706-0)
Supplement: Supplementary file 3 — Description of Additional Supplementary Files [file 42003_2022_3706_MOESM3_ESM.pdf]

Description of Additional Supplementary Files

File name: Supplementary Data 1
Description: List of specimens used in the study, measurements, and references..
